# Major surgical postoperative complications and survival in breast cancer: Swedish population-based register study in 57 152 women

**DOI:** 10.1093/bjs/znac275

**Published:** 2022-08-05

**Authors:** Jana de Boniface, Robert Szulkin, Anna L V Johansson

**Affiliations:** Department of Surgery, Capio St Göran’s Hospital, Stockholm, Sweden; Department of Molecular Medicine and Surgery, Karolinska Institutet, Stockholm, Sweden; SDS Life Science, Danderyd, Sweden; Department of Medical Epidemiology and Biostatistics, Karolinska Institutet, Stockholm, Sweden; Department of Medical Epidemiology and Biostatistics, Karolinska Institutet, Stockholm, Sweden; Cancer Registry of Norway, Oslo, Norway

## Abstract

**Background:**

Postoperative complications may activate prometastatic systemic pathways through tissue damage, wound healing, infection, and inflammation. Postoperative complications are associated with inferior survival in several types of cancer. The aim was to determine the association between postoperative complications and survival in breast cancer.

**Methods:**

This population-based cohort included women operated for T1–3 N0–3 M0 invasive breast cancer in Sweden from 2008 to 2017. Only major surgical postoperative complications leading to readmission and/or reoperation within 30 days were considered. Main outcomes were overall survival (OS) and breast cancer-specific survival (BCSS). Prospectively collected nationwide register data were used. Multivariable Cox models were adjusted for clinical and socioeconomic confounders and co-morbidity.

**Results:**

Among 57 152 women, major surgical postoperative complications were registered for 1854 patients. Median follow-up was 6.22 (0.09–11.70) years. Overall, 9163 patients died, and 3472 died from breast cancer. Major surgical postoperative complications were more common after mastectomy with or without immediate reconstruction (7.3 and 4.3 per cent respectively) than after breast-conserving surgery (2.3 per cent). Unadjusted 5-year OS and BCSS rates were 82.6 (95 per cent c.i. 80.8 to 84.5) and 92.1 (90.8 to 93.5) per cent respectively for women with a major surgical postoperative complication, and 88.8 (88.6 to 89.1) and 95.0 (94.8 to 95.2) per cent for those without a complication (*P* < 0.001). After adjustment, all-cause and breast cancer mortality rates remained higher after a major surgical postoperative complication (OS: HR 1.32, 95 per cent c.i. 1.15 to 1.51; BCSS: HR 1.31, 1.04 to 1.65). After stratification for type of breast surgery, this association remained significant only for women who had mastectomy without reconstruction (OS: HR 1.41, 1.20 to 1.66; BCSS: HR 1.36, 1.03 to 1.79).

**Conclusion:**

Major surgical postoperative complications are associated with inferior survival, especially after mastectomy. These results underline the importance of surgical de-escalation.

## Teaser

Postoperative complications after cancer surgery may contribute to a prometastatic environment and inferior oncological outcomes. In this large population-based study, major surgical complications were associated with worse cancer-specific and overall survival, especially in patients undergoing mastectomy for breast cancer.

## Introduction

Postoperative complications (POCs) may occur after any operation and include non-infectious (such as bleeding, seroma, delayed wound healing) and/or infectious complications. In a prospective cohort study^1^ published by the GlobalSurg Collaborative, 30-day major complications (corresponding to Clavien–Dindo grade III–V) after breast cancer surgery occurred in 5.9 per cent and any complication in 36.1 per cent. The reporting of frequency and severity of POCs is dependent on high completeness of registration and the implementation of clear definitions. Both these factors are, however, prone to significant heterogeneity, resulting in wide variability in reported complication rates, in particular in retrospective settings.

Surgical tissue trauma and the wound healing process activate multiple pathways relevant in the context of cancer surgery because they are associated with a prometastatic environment. A systemic stress response exerts immunosuppressive effects, and proinflammatory and proangiogenic pathways may promote tumour cell growth, adhesion, and metastatic potential^2^. In a small randomized trial^3^, the perioperative inhibition of cyclo-oxygenase-2 and β-adrenergic signalling reduced the activity of prometastatic and proinflammatory factors and affected tumour-infiltrating monocytes and B cells in patients with breast cancer.

POCs are thought to add to, prolong, and accentuate these processes. Postoperative infection may enhance the adhesion and survival of circulating tumour cells through the activation of toll-like receptors (TLRs), and thus promote metastases^4^. POCs may also delay postoperative systemic treatment that is specifically aimed at eradicating residual tumour cells^5,6^. An adverse effect of POCs on oncological outcomes has been shown in colorectal, gastric, lung, and head and neck cancer^7^. In breast cancer, a recent systematic review and meta-analysis^8^ included 10 cohort studies, 4 of which reported an association between POCs and outcome. Owing to a high degree of heterogeneity and limitations in both follow-up time and population sizes, the results were deemed inconclusive and further research was called for. In breast cancer surgery, POCs are significantly more common after mastectomy than after breast-conserving surgery (BCS). In a joint analysis of the prospective iBRA-2 and TeaM cohort studies^9^, major complications occurred in 2.1 per cent of women receiving therapeutic mammaplasty, whereas mastectomy with and without immediate reconstruction (IBR) resulted in major complication rates of 14.4 and 5.0 per cent respectively. Whether this observation has potential implications for the recently reported superior survival after breast conservation and that of impaired survival after breast reconstruction has not yet been investigated.

The aim of this analysis was to determine the association between major surgical postoperative complications (msPOCs) and survival outcomes using large-scale prospectively collected data from population-based registers, taking into consideration confounding by demographics, socioeconomic status, type of breast and axillary surgery, disease and treatment characteristics, and co-morbidity.

## Methods

### Study population

Women diagnosed with invasive breast cancer from 1 January 2008 to 31 December 2017, registered in the Swedish National Quality Register for Breast Cancer (NKBC), were included in this nationwide cohort study using prospectively collected register data. The NKBC includes detailed clinical information on all patients with breast cancer diagnosed in Sweden, and is deemed 98–99 per cent complete, registering nearly all new breast cancer diagnoses in the country^10^. Information on patient age and region of residence, date of diagnosis, date and type of surgery, tumour characteristics, and neoadjuvant and adjuvant treatment was extracted. Women were eligible if they had a known date and type of surgery, and available data on tumour size and planned or administered radiotherapy, but did not have locally advanced tumours (T4) or distant metastases.

The cohort was individually linked to Swedish population-based registers via the unique Swedish personal identification number assigned to all residents. Information was extracted from the National Patient Registers at the National Board of Health and Welfare on inpatient admissions and outpatient visits from 2008 to 2017, including dates of admission and discharge, diagnosis, and procedure codes. Date and cause of death was obtained from the Cause of Death Register, and complemented with information from the Total Population Register at Statistics Sweden. Death owing to breast cancer was defined as death with an ICD-10 code C50 as the registered cause of death and was available until September 2019.

Information was obtained from the Longitudinal Integrated Database for Health Insurance and Labour Market Studies at Statistics Sweden on country of birth, highest educational level (9 years or less (primary), 10–13 years (secondary), or more than 13 years (tertiary)), and family income (low (Q1: 0–25 per cent), middle (Q2–Q3: >25–75 per cent), or high (Q4: >75–100 per cent)). Educational level and income were obtained from 1 year before cancer diagnosis, and income was adjusted for inflation over the study interval. If diagnosis occurred in 2008, information on educational level and income in 2008 was used.

Both main and contributing diagnoses of conditions listed in the Royal College of Surgeons Charlson Co-morbidity Index (CCI), and registered in the National Patient Registers between 2008 and 2017 and within 12 months before start of treatment, were used (*Table S1*). If diagnosis occurred in 2008, co-morbidity in 2008 was considered.

For women with bilateral tumours, the side with the larger tumour and/or more advanced nodal category was selected, and second or more recorded breast tumours in the same individual were excluded.

### Major surgical postoperative complications

Major surgical postoperative complication (msPOC) was defined as the occurrence of at least one prespecified diagnostic or intervention code regarding bleeding or wound complication (T810, T811, T817, HWD00, HWE00, HWA00, T813, HWF00), infection (T857, T814, HWB00, HWC00), and/or unspecified complications (T854, T856, T858, T859, T812, T815, T818, T818W, T819, HWW99, T889) registered in association with the index inpatient episode or a new inpatient episode (readmission) within 30 days of first surgery. ICD-10 was used (*Table S2*).

### Disease characteristics and treatment

Tumour size (T) and nodal status (N) were defined according to the eighth edition of the AJCC Cancer Staging Manual, and based on pathology after primary surgery, and on clinical and/or radiological assessment if neoadjuvant treatment was given. Tumour biology was based on pretreatment core needle biopsy if neoadjuvant treatment was administered, and on the surgical specimen otherwise, and included oestrogen receptor (ER), progesterone receptor (PR), and human epidermal growth factor receptor 2 (HER2) amplification. ER and PR were considered negative if less than 10 per cent. HER2 amplification was confirmed by an immunohistochemial score of 3+ or by *in situ* hybridization, which was carried out in the event of a score of 2 +. Hormone receptor-positive (HR+) tumours were ER+ and/or PR+, and hormone receptor-negative (HR−) tumours were ER− and PR−. Subtypes were classified as HR + HER2−, HR + HER2+, HR− HER2+, and HR− HER2−.

Primary treatment was defined as primary surgery or neoadjuvant systemic treatment. Type of final surgical treatment was categorized as BCS or mastectomy with or without IBR, and final type of axillary surgery as sentinel lymph node biopsy (SLNB) or axillary lymph node dissection (ALND). If performed for the same ipsilateral breast cancer diagnosis, SLNB followed by ALND was classified as ALND, and BCS followed by completion mastectomy as mastectomy with or without IBR.

### Ethical considerations

The study was approved by the regional Ethical Review Authority in Stockholm (2017/2493-31) and carried out in accordance with the ethical standards of the Helsinki Declaration of 1975. No specific informed consent was obtained because general consent for the use of personal data was given when accepting registration in the NKBC. Registration in other national registers used is mandatory by law and does not require consent.

### Statistical analysis

Descriptive frequencies of disease and treatment characteristics by msPOC (no msPOC *versus* at least 1 msPOC within 30 days) were calculated. To assess the impact of preoperative patient age, demographic and socioeconomic data, primary treatment, and type of breast and axillary surgery on risk of msPOC, ORs with 95 per cent confidence intervals were estimated using logistic regression. OS was investigated as time to death from any cause, and breast cancer-specific survival (BCSS) as time to death from breast cancer. Time of follow-up was from 30 days after surgery until date of death or end of study (30 September 2019), whichever came first. Five- and 10-year OS and BCSS rates were estimated using the Kaplan–Meier method, and presented for patients with and without msPOCs. HRs with 95 per cent confidence intervals were estimated using stratified Cox regression models, comparing patients with and without msPOCs. Models were adjusted in a stepwise fashion for patient characteristics (age, year of first surgery, region of residence), disease characteristics (TNM stage, grade, histological type, subtype), type of breast and axillary surgery, socioeconomic data (education, family income, country of birth), and CCI score to assess confounding. The proportional hazards assumption was evaluated with tests based on Schoenfeld residuals. To account for non-proportionality in TNM stage, grade, and histological type and subtype, the Cox models were stratified by these variables, meaning that separate baseline hazards were used for each level of these factors, yet common effects of the remaining co-variates were estimated. For sensitivity analyses, patients who had more than one operation for the index breast cancer were excluded, and thereafter also patients with tumours larger than 2 cm. In a second step, HRs for women with and without msPOCs were estimated by type of breast surgery. Flexible parametric survival models were used to estimate the HR of msPOCs as a smooth function of time, by use of a restricted cubic spline with five degrees of freedom for the baseline hazard and a spline with three degrees of freedom for the time-dependent effect^11^.

All tests were two-sided, and the significance level was 5 per cent. Analyses were performed in R version 4.1.0 (R Foundation for Statistical Computing, Vienna, Austria).

## Results

After linkages, women with an unclear surgery date (8), reused personal identification number (52) or recorded death before breast cancer diagnosis (1), and those who died within 30 days of surgery (24) were excluded, along with second or more recorded breast tumours in the same individual (1472), leaving a total of 57 152 women for the final analysis (*Fig. S1*).

Overall, 1993 msPOCs leading to readmission and/or reoperation were registered during a 30-day interval after first surgery in 1854 (3.2 per cent) of 57 152 women. More than one type of complication was registered for 107 patients. Bleeding complications (1282) most commonly occurred during the same hospital admission as the first operation (941, 73.4 per cent), whereas infectious complications (536) more commonly led to a later readmission (496, 92.5 per cent). Corresponding figures for reoperation (775) were 568 (77.3 per cent) and 207 (26.7 per cent). Some unspecified msPOCs (175) were registered both during the first hospital stay (59, 33.7 per cent) or a later readmission (116, 66.3 per cent).

Women with msPOCs more commonly had larger tumours (*P* < 0.001), node-positive disease (*P* < 0.001), HER2-positive subtypes (*P* = 0.011), high-grade tumours (*P* < 0.001), and had received preoperative systemic therapy (*P* < 0.001). The youngest and oldest age groups were over-represented in the msPOC group (*P* < 0.001), but median age did not differ. Mastectomy with or without IBR (*P* < 0.001) and ALND (*P* < 0.001) were more common in women with msPOCs. Having msPOCs was significantly associated with region of residence (*P* < 0.001), and weakly associated with lower income (*P* = 0.044), but not with educational level or country of birth. Women in the msPOC group more commonly also had more co-morbidities (*P* < 0.001) (*Table S3*).

The rate of msPOCs was significantly associated with the extent of breast surgery (BCS 2.3 per cent; mastectomy 4.3 per cent; mastectomy + IBR 7.3 per cent; *P* < 0.001). ALND was associated with a higher msPOC rate than SLNB in women undergoing BCS (3.7 *versus* 2.0 per cent). This finding was less pronounced after mastectomy without (4.4 *versus* 4.1 per cent) or with (7.4 *versus* 7.1 per cent) IBR.

After adjustments, type of breast and axillary surgery, region of residence, and co-morbidity were independently associated with msPOCs within 30 days. The risk of msPOCs was 1.68 (95 per cent c.i. 1.50 to 1.87) times higher after mastectomy without IBR and 2.80 (2.24 to 3.47) times higher after mastectomy with IBR than after BCS. ALND increased the risk of msPOCs by 38 per cent (OR 1.38, 95 per cent c.i. 1.24 to 1.54) compared with SLNB only (*Table S4*).

**Fig. 1:**
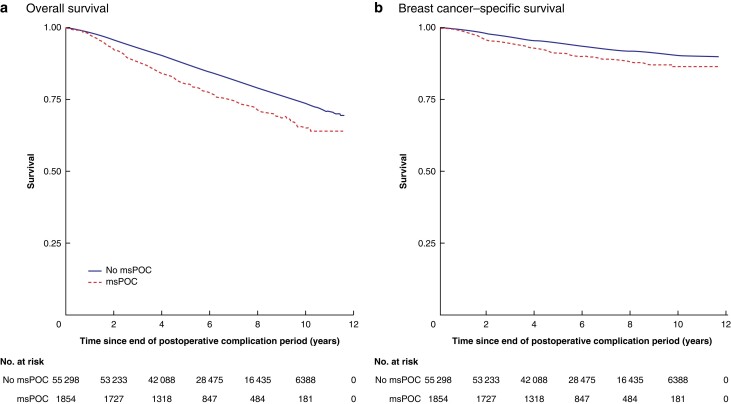
Unadjusted Kaplan–Meier survival curves for individuals with and without at least one major surgical postoperative complication **a** Overall survival and **b** breast cancer-specific survival, msPOC, major surgical postoperative complication. **a**,**b**  *P **<*** 0.001 (log rank test).

After a median follow-up of 6.22 years, 9163 deaths had occurred and 3472 patients had died from breast cancer. Five- and 10-year OS rates were 88.8 (95 per cent c.i. 88.6 to 89.1) and 76.4 (75.9 to 77.0) per cent respectively for women without msPOCs, compared with 82.6 (80.8 to 84.5) and 68.9 (65.9 to 72.1) per cent for those with msPOCs. Five- and 10-year BCSS rates were 95.0 (94.8 to 95.2) and 91.4 (91.1 to 91.7) per cent respectively for women without msPOCs, compared with 92.1 (90.8 to 93.5) and 88.1 (86.0 to 90.1) per cent for those with msPOCs. Unadjusted survival rates (OS and BCSS) were significantly worse in the msPOC group (*P* < 0.001) (*Fig. 1*).

After stepwise adjustment for age, demographic variables, disease characteristics, treatment, socioeconomic status, and co-morbidity, the occurrence of msPOCs was still significantly associated with higher rates of all-cause mortality (HR 1.32, 95 per cent c.i. 1.15 to 1.51) and breast cancer mortality (HR 1.31, 1.04 to 1.65). Subgroup analysis showed that the association with death from any cause remained significant in separate analysis for bleeding or wound complications (HR 1.35, 1.15 to 1.59) and infectious complications (HR 1.37, 1.08 to 1.75). In terms of breast cancer mortality, however, only bleeding and wound complications (HR 1.51, 1.15 to 1.99), and not infections (HR 0.95, 0.61 to 1.49), remained significant (*Table 1*).

**Table 1:** Hazard ratios for all-cause death and breast cancer death after a major surgical postoperative complication with stepwise adjustments for patient and disease characteristics, treatment, socioeconomic status, and Charlson Co-morbidity Index, with separate subgroup analyses for bleeding and wound complications or infections only

	HR						
	Model 0*	Model 1†	Model 2‡	Model 3§	Model 4¶	Model 5#	Model 6**
**All-cause death**							
No msPOC	1.00 (reference)	1.00 (reference)	1.00 (reference)	1.00 (reference)	1.00 (reference)	1.00 (reference)	1.00 (reference)
Any msPOC	1.47 (1.33, 1.63)	1.40 (1.27, 1.55)	1.44 (1.28, 1.63)	1.40 (1.24, 1.59)	1.39 (1.23, 1.58)	1.40 (1.23, 1.59)	1.32 (1.15, 1.51)
Bleeding or wound complication	1.44 (1.28, 1.63)	1.34 (1.18, 1.51)	1.50 (1.28, 1.74)	1.44 (1.23, 1.68)	1.46 (1.25, 1.71)	1.44 (1.23, 1.68)	1.35 (1.15, 1.59)
Infection	1.66 (1.39, 1.97)	1.60 (1.34, 1.90)	1.60 (1.29, 1.99)	1.56 (1.25, 1.94)	1.53 (1.22, 1.91)	1.49 (1.19, 1.86)	1.37 (1.08, 1.75)
**Breast cancer death**							
No msPOC	1.00 (reference)	1.00 (reference)	1.00 (reference)	1.00 (reference)	1.00 (reference)	1.00 (reference)	1.00 (reference)
Any msPOC	1.52 (1.30, 1.78)	1.46 (1.24, 1.71)	1.45 (1.18, 1.78)	1.42 (1.15, 1.75)	1.47 (1.17, 1.84)	1.37 (1.11, 1.71)	1.31 (1.04, 1.65)
Bleeding or wound complication	1.42 (1.17, 1.73)	1.36 (1.11, 1.65)	1.61 (1.24, 2.08)	1.56 (1.21, 2.02)	1.61 (1.25, 2.08)	1.60 (1.24, 2.07)	1.51 (1.15, 1.99)
Infection	1.72 (1.30, 2.27)	1.62 (1.23, 2.14)	1.27 (0.87, 1.84)	1.25 (0.86, 1.82)	1.13 (0.76, 1.68)	1.09 (0.73, 1.63)	0.95 (0.61, 1.49)

Values in parentheses are 95 per cent confidence intervals.

* Unadjusted model.

† Adjusted for age, year of surgery, and region of residence at diagnosis.

‡ Adjusted for same variables as model 1 plus stratified by prognostic group (TNM stage), Nottingham histological grade, histological subtype, hormone receptor (oestrogen receptor, progesterone receptor) and human epidermal growth factor receptor 2 (HER2) amplification status.

§ Adjusted for same variables as model 2 plus type of breast and axillary surgery.

¶ Adjusted for same variables as model 3 plus family income and highest education level.

# Adjusted for same variables as model 4 plus Charlson Co-morbidity Index score 1 year before surgery.

** Adjusted for same variables as model 5 plus HER2*-*targeted treatment and chemotherapy, stratified by adjuvant radiotherapy and endocrine treatment. msPOC, major surgical postoperative complication.

To evaluate a potential effect of reoperation for an oncological indication (positive margins), sensitivity analyses were undertaken with exclusion of 4812 patients who had undergone more than one operation for breast cancer. The results showed essentially similar HRs for the association between msPOCs and higher rates of all-cause mortality (HR 1.32, 1.14 to 1.51) and breast cancer mortality (HR 1.25, 0.98 to 1.60) in the multivariable model. To further elucidate the specific situation for patients with small tumours (T1) who should be potential candidates for BCS, a subgroup analysis excluding 23 848 women who had more than one breast cancer operation and/or tumours larger than 2 cm was performed. The corresponding HRs remained nearly unchanged for all-cause mortality (HR 1.33, 1.07 to 1.64) and breast cancer death (HR 1.41, 0.90 to 2.22).

In analyses of the associations between msPOCs and mortality stratified by type of breast surgery, msPOCs increased the risk of death from all causes (HR 1.41, 1.20 to 1.66) and breast cancer death (HR 1.36, 1.03 to 1.79) in the subgroup of women undergoing mastectomy without IBR (*Table 2*). For women undergoing BCS, HRs were not significantly increased (OS: HR 1.17, 95 per cent c.i. 0.91 to 1.50; BCSS: HR 1.23, 0.80 to 1.91). For mastectomy with reconstruction, only one death had occurred and no association was found.

**Table 2:** All-cause and breast cancer-specific death by interaction between type of breast surgery and at least one major surgical postoperative complication

	No. of deaths from any cause	HR	No. of breast cancer deaths	HR
**Breast-conserving surgery**				
No msPOC	2107	1.00 (reference)	629	1.00 (reference)
Any msPOC	73	1.17 (0.91, 1.50)	25	1.23 (0.80-1.91)
**Mastectomy**				
No msPOC	3265	1.00 (reference)	1266	1.00 (reference)
Any msPOC	192	1.41 (1.20, 1.66)	71	1.36 (1.03, 1.79)
**Mastectomy + IBR**				
No msPOC	49	1.00 (reference)	27	1.00 (reference)
Any msPOC	1	0.38 (0.05, 2.84)	1	0.63 (0.08, 4.99)

Values in parentheses are 95 per cent confidence intervals. msPOC, major surgical postoperative complication; IBR, immediate breast reconstruction.

*Figure S2* shows the adjusted HR over time since surgery for women with *versus* without msPOCs. The increased mortality rate owing to breast cancer was highest close to diagnosis and lasted for up to 4 years after surgery. For death from any cause, the increased mortality rate extended over nearly 6 years after surgery.

## Discussion

In this comprehensive analysis of a large prospectively maintained population-based register, the occurrence of at least one msPOC was independently associated with worse survival, especially in women undergoing mastectomy. More extensive surgery was significantly associated with a higher risk of msPOCs.

Ten studies were included in a systematic review and meta-analysis by Savioli *et al.*^8^, most of which were retrospective cohort series of varying size. Two included studies were population-based. Pedersen *et al.*^12^ used a large population from national Danish registers and could not find any association between postoperative bleeding complications within 14 days and oncological outcome. de Glas *et al.*^13^ analysed a subset of women aged 65 years and older from the National Cancer Registry in the Netherlands in terms of a wide array of complications, including minor surgical complications and medical complications. It was concluded that, even though the older patient population was at higher risk of experiencing POCs, these did not contribute to inferior survival. Several reports^14–18^ included in the meta-analysis focused on women receiving breast reconstruction. Furthermore, there was significant heterogeneity in measured outcomes, which comprised, among others, postoperative fever, wound complications, surgical and medical complications, reoperation for bleeding, and acute infection, leading to event rates ranging from 2.5 to 45 per cent^12–21^. Owing to limited populations sizes in all but the Danish and Dutch register studies, adjustment for potential confounders was limited. In contrast, the present population, representing the largest sample so far, was not limited to specific surgical interventions, ages or disease stages, allowing the integration of important confounders in multivariable modelling, even though statistical power was impaired in sensitivity analyses.

There is a common clinical belief that mastectomy may be the ideal surgical intervention for the frail and elderly, avoiding the risk of reoperation for positive margins and potentially the need for adjuvant radiotherapy. The association between older age and having mastectomy without subsequent radiotherapy was described in a recent publication^22^ from the same database. Unfortunately, no national registers record the degree of frailty at a given time point or the factors needed to evaluate the state of frailty, such as unintentional weight loss in the past year, low grip strength, self-reported exhaustion, slow walking speed, and low physical activity. Considering the increasing prevalence of frailty with ageing, one should, however, be aware that a mastectomy may not only put the older and/or frail patient at a higher risk of msPOCs but also at a higher risk of death.

Women with breast cancer undergoing breast reconstruction are commonly younger and healthier, and have a higher socioeconomic status than their counterparts not receiving reconstruction^23,24^. Women who undergo breast reconstruction tend to have better survival than those with comparable disease and treatment characteristics, suggesting a selection bias present at the time of surgical planning^25–27^. It has been reported previously, however, that complications after IBR are associated with worse recurrence and survival rates^18,28^. In the present analysis, mastectomy with IBR carried the highest risk of msPOCs, but survival outcomes were at least equivalent to those after BCS after adjustments for confounding factors.

A major player in the context of surgical trauma and systemic prometastastic processes is the family of TLRs^4^. TLRs are present within tumours but also in barrier tissues and on haematopoietic and immunological cells throughout the body. Activation occurs through pathogen-associated molecular patterns, which include bacterial infection among others, but also through non-infectious inflammation induced by damage-associated molecular patterns, which are typically present in the context of surgical procedures. As breast cancer cells carry their own TLRs, their metastatic potential may be augmented through surgery^29,30^. This is important considering the circulating tumour cells (CTCs) that are frequently found in breast cancer. CTCs rarely succeed in establishing metastases, but their presence remains a prognostic factor, and their survival may be propagated by the systemic consequences of POCs^31^. In the present analysis, both bleeding and infectious msPOCs were associated with survival outcomes. Future prospective trials should evaluate whether perioperative immunomodulatory drug administration has the potential to improve oncological outcomes.

The strength of the present analysis is its high external validity owing to the national population-based setting with extensive validated data and complete follow-up. It is essential to integrate potential confounders affecting both survival and the risk of msPOCs into analyses in order not to overestimate the importance of complications. Some potential confounders, such as smoking and BMI, were not available, but may be expected to be partly reflected by socioeconomic data. The fact that regional differences in msPOC rates were observed may suggest reporting bias, but could also represent the fact that the region with the highest risk of msPOCs has the highest immediate reconstruction rate. In addition, the Charlson Co-morbidity Index does not include all potentially relevant co-morbid conditions, leading to possible underestimation of co-morbidity and risk of residual confounding. To address this limitation, data on inpatient as well as outpatient care, using both main and contributing diagnoses, were included. Another potential limitation is that no data on delay in initiation of adjuvant treatment were available. Patients with msPOCs had more severe disease characteristics, and it cannot be excluded that this had an impact on survival outcomes. In addition, frailty, of major significance especially in the older population, could not be assessed. Finally, no reliable information on recurrences was available, precluding the analysis of disease-free survival between the groups.

## Funding

This work was funded by the Swedish Breast Cancer Association (Bröstcancerförbundet). J.d.B. is supported by a Junior Clinical Investigator Award from the Swedish Cancer Society (Cancerfonden) (CAN 2017/1036). A.L.V.J. is supported by a Research Starting Grant from the Swedish Research Council (Vetenskapsrådet) (2021–01657). The funding organization had no role in the design and conduct of the study; collection, management, analysis, and interpretation of the data; preparation, review, or approval of the manuscript; and the decision to submit the manuscript for publication.

## Supplementary Material

znac275_Supplementary_Data
