# Supplementary material for: Major surgical postoperative complications and survival in breast cancer: Swedish population-based register study in 57 152 women
Source: Br J Surg. 2022 Aug 5;109(10):977–83. doi: 10.1093/bjs/znac275 (PMC10364684; doi:10.1093/bjs/znac275)
Supplement: znac275_Supplementary_Data [file znac275_supplementary_data.zip › Supplementary_Figure_1.docx]

**Figure S1.** CONSORT flow diagram

**Data extraction from NKBC 2008-2017**

**N=66490 invasive breast cancers**

**Exclusions before register linkages:**

- No breast surgery performed, N=134
- Type of breast surgery not reported, N=4917
- Reported as having distant metastasis at diagnosis and/or no surgery planned, N=162
- Locally advanced tumors (T4), N=572
- Clinical (in neoadjuvant cases) or pathological (in primary surgery cases) T stage not reported, N=1141
- Non-invasive breast cancer reported incorrectly to the register, N=1
- No registered information on planned or given adjuvant radiotherapy, N=854

**Exclusions after register linkages:**

- Second or more recorded breast cancers in NKBC, N=1472
- 8 deviating date of surgery (surgery date unclear)
- 52 reused personal identification numbers (no register linkage possible)
- 1 recorded death prior to diagnosis date
- 24 deaths within 30 days of surgery

**Included in survival analysis**

**N=57152**

**N=58709 breast cancers (in 57237 women)**

**Linkage to Patient Registers, Cause of Death Register and LISA database**

**INCLUSION CRITERIA:**

**Women with first recorded breast cancer 2008-2017, T1-T3, any N, M0, with known date of surgery**

**N=57152**
