# Supplementary material for: Major surgical postoperative complications and survival in breast cancer: Swedish population-based register study in 57 152 women
Source: Br J Surg. 2022 Aug 5;109(10):977–83. doi: 10.1093/bjs/znac275 (PMC10364684; doi:10.1093/bjs/znac275)
Supplement: znac275_Supplementary_Data [file znac275_supplementary_data.zip › Supplementary_Tables.docx]

**Table S1.** ICD-10 codes based on the Royal College of Surgeons’ adaptation of the Charlson Comorbidity Index

| **Disease category** | **Weight** | **ICD-10** |
| --- | --- | --- |
| Diabetes mellitus | 1 | E10, E11, E12, E13, E14 |
| Dementia | 1 | F00, F01, F02, F03, G30, G31, A81, F05 |
| Cerebrovascular Disease | 1 | G45, G46, I60, I61, I62, I63, I64, I65, I66, I67, I68, I69 |
| Congestive Heart Failure | 1 | I11, I42, I43, I50, I51 |
| Congestive Heart Failure or Renal Disease * | 1 | I13 |
| Myocardial Infarction | 1 | I21, I22, I23 |
| Congestive Heart Failure or Myocardial Infarction * | 1 | I25 |
| Chronic Pulmonary Disease | 1 | I26, I27, J40, J41, J42, J43, J44, J45, J46, J47, J60, J61, J62, J63, J64, J65, J66, J67, J68, J70 |
| Peripheral Vascular Disease | 1 | I70, I71, I72, I73, I77, K55, Z95 |
| Rheumatological Disease | 1 | M05, M06, M09, M32, M33, M34, M35, M36, M12, M31 |
| Any malignancy | 2 | C00, C01, C02, C03, C04, C05, C06, C07, C08, C09, C10, C11, C12, C13, C14, C15, C16, C17, C18, C19, C20, C21, C22, C23, C24, C25, C26, C30, C31, C32, C33, C34, C37, C38, C39, C40, C41, C43, C45, C46, C47, C48, C49, C50, C51, C52, C53, C54, C55, C56, C57, C58, C60, C61, C62, C63, C64, C65, C66, C67, C68, C69, C70, C71, C72, C73, C74, C75, C76, C80, C81, C82, C83, C84, C85, C88, C90, C91, C92, C93, C94, C95, C96, C97 |
| Hemiplegia or paraplegia | 2 | G81, G82, G83, G11 |
| Renal Disease | 2 | I12, N01, N03, N05, N07, N08, N18, N19, N25, Z49, N17, Z99 |
| Renal Disease or Liver Disease * | 2 | Z94 |
| Liver Disease | 3 | B18, I85, K70, K71, K76, I86, I98, K72 |
| AIDS/HIV | 6 | B20, B21, B22, B24 |
| Metastatic solid tumor | 6 | C77, C78, C79 |

* Due to ICD-10 being included as 3-digit codes in the patient register, it was not possible to separate these diseases.

| **ICD-10 code** | **Definition** |
| --- | --- |
| *Bleeding complication* | |
| T810 | Haemorrhage and haematoma complicating a procedure, not elsewhere classified |
| T811 | Shock during or resulting from a procedure, not elsewhere classified |
| T817 | Vascular complications following a procedure, not elsewhere classified |
| T813 | Disruption of operation wound, not elsewhere classified |
| HWD00 | Reoperation for superficial haemorrhage after breast surgery |
| HWE00 | Reoperation for deep haemorrhage after breast surgery |
| HWA00 | Reoperation for wound dehiscence after breast surgery |
| HWF00 | Reoperation for insufficient suture after breast surgery |
| *Infectious complication* | |
| T857 | Infection and inflammatory reaction due to other internal prosthetic devices, implants and grafts |
| T814 | Infection following a procedure, not elsewhere classified |
| HWB00 | Reoperation for superficial infection after breast surgery |
| HWC00 | Reoperation for deep infection after breast surgery |
| *Other complication* | |
| T854 | Mechanical complication of breast prosthesis and implant |
| T856 | Mechanical complication of other specified internal prosthetic devices, implants and grafts |
| T858 | Other complications of internal prosthetic devices, implants and grafts, not elsewhere classified |
| T859 | Unspecified complication of internal prosthetic device, implant and graft |
| T812 | Accidental puncture and laceration during a procedure, not elsewhere classified |
| T815 | Foreign body accidentally left in body cavity or operation wound following a procedure |
| T818 | Disruption of operation wound, not elsewhere classified |
| T819 | Unspecified complication of procedure |
| T889 | Complication of surgical and medical care, unspecified |
| HWW99 | Other reoperation on breast gland, unspecified |

**Table S2**. Disease codes from the International Statistical Classification of Diseases and Related Health Problems, Tenth Revision (ICD-10) and surgical intervention codes used to define surgical postoperative complications.

**Table S3.** Patient, treatment and disease characteristics

|  | **No surgical postoperative complication**  **N=55298** | **Surgical postoperative complication**  **N=1854** | **Overall**  **N=57152** | ***P* value** |
| --- | --- | --- | --- | --- |
| **Follow-up* (years)** | 6.24 (0.09-11.70) | 5.74 (0.11-11.67) | 6.22 (0.09-11.70) | <0.001 |
| **Year of surgery** |  |  |  | 0.308 |
| 2008-2009 | 10441 (18.9) | 337 (18.2) | 10778 (18.9) |  |
| 2010-2011 | 12246 (22.1) | 381 (20.6) | 12627 (22.1) |  |
| 2012-2013 | 12656 (22.9) | 447 (24.1) | 13103 (22.9) |  |
| 2014-2015 | 13004 (23.5) | 458 (24.7) | 13462 (23.6) |  |
| 2016-2017 | 6951 (12.6) | 231 (12.5) | 7182 (12.6) |  |
| **Age (years) at diagnosis*** | 63.0 (19-99) | 63.5 (22-95) | 63.0 (19-99) | 0.592 |
| **Age group (years)** |  |  |  | <0.001 |
| <40 | 2133 (3.9) | 85 (4.6) | 2218 (3.9) |  |
| 40-49 | 7877 (14.2) | 301 (16.2) | 8178 (14.3) |  |
| 50-64 | 19480 (35.2) | 603 (32.5) | 20083 (35.1) |  |
| 65-74 | 16934 (30.6) | 494 (26.6) | 17428 (30.5) |  |
| 75 and more | 8874 (16.0) | 371 (20.0) | 9245 (16.2) |  |
| **Tumour stage^** |  |  |  | <0.001 |
| T1 | 35400 (64.0) | 996 (53.7) | 36396 (63.7) |  |
| T2 | 17119 (31.0) | 729 (39.3) | 17848 (31.2) |  |
| T3 | 2779 (5.0) | 129 (7.0) | 2908 (5.1) |  |
| **Nodal stage^** |  |  |  | <0.001 |
| N0 | 36443 (65.9) | 1059 (57.1) | 37502 (65.5) |  |
| N1 | 12785 (23.1) | 528 (28.5) | 13313 (23.3) |  |
| N2 | 2885 (5.2) | 120 (6.5) | 3005 (5.3) |  |
| N3 | 1346 (2.4) | 76 (4.1) | 1422 (2.5) |  |
| Missing | 1839 (3.3) | 71 (3.8) | 1910 (3.3) |  |
| **Region of residence** |  |  |  | <0.001 |
| Stockholm/Gotland | 12151 (22.0) | 491 (26.5) | 12642 (22.1) |  |
| Uppsala/Örebro | 11907 (21.5) | 339 (18.3) | 12246 (21.4) |  |
| North | 4778 (8.6) | 203 (10.9) | 4981 (8.7) |  |
| South | 10518 (19.0) | 296 (16.0) | 10814 (18.9) |  |
| Southeast | 5684 (10.3) | 175 (9.4) | 5859 (10.3) |  |
| West | 10260 (18.6) | 350 (18.9) | 10610 (18.6) |  |
| **Primary treatment** |  |  |  | <0.001 |
| Surgery | 52434 (94.8) | 1715 (92.5) | 54149 (94.7) |  |
| NAT | 2864 (5.2) | 139 (7.5) | 3003 (5.3) |  |
| **Breast surgery** |  |  |  | <0.001 |
| BCS | 32403 (58.6) | 767 (41.4) | 33170 (58.0) |  |
| Mastectomy | 21418 (38.7) | 970 (52.3) | 22388 (39.2) |  |
| Mastectomy + IBR | 1477 (2.7) | 117 (6.3) | 1594 (2.8) |  |
| **Axillary surgery** |  |  |  | <0.001 |
| SLNB | 36198 (65.5) | 993 (53.6) | 37191 (65.1) |  |
| ALND | 17759 (32.1) | 797 (43.0) | 18556 (32.5) |  |
| Missing | 1341 (2.4) | 64 (3.5) | 1405 (2.5) |  |
| **Histological tumour type** |  |  |  | 0.337 |
| Ductal | 41626 (75.3) | 1355 (73.1) | 42981 (75.2) |  |
| Lobular | 7114 (12.9) | 249 (13.4) | 7363 (12.9) |  |
| Other invasive | 3615 (6.5) | 107 (5.8) | 3722 (6.5) |  |
| Missing | 2943 (5.3) | 143 (7.7) | 3086 (5.4) |  |
| **Nottingham histological grade^** |  |  |  | <0.001 |
| Grade 1 | 11065 (20.0) | 285 (15.4) | 11350 (19.9) |  |
| Grade 2 | 26214 (47.4) | 876 (47.2) | 27090 (47.4) |  |
| Grade 3 | 14580 (26.4) | 532 (28.7) | 15112 (26.4) |  |
| Missing | 3439 (6.2) | 161 (8.7) | 3600 (6.3) |  |
| **Subtype^** |  |  |  | 0.017 |
| HR+HER2- | 37186 (67.2) | 1237 (66.7) | 38423 (67.2) |  |
| HR+HER2+ | 4160 (7.5) | 158 (8.5) | 4318 (7.6) |  |
| HR-HER2+ | 1408 (2.5) | 67 (3.6) | 1475 (2.6) |  |
| HR-HER2- | 3087 (5.6) | 98 (5.3) | 3185 (5.6) |  |
| Missing | 9457 (17.1) | 294 (15.9) | 9751 (17.1) |  |
| **Radiotherapy** |  |  |  | <0.001 |
| Yes | 39464 (71.4) | 1123 (60.6) | 40587 (71.0) |  |
| No | 15834 (28.6) | 731 (39.4) | 16565 (29.0) |  |
| **Chemotherapy^+^** |  |  |  | <0.001 |
| Yes | 17520 (31.7) | 683 (36.8) | 18203 (31.9) |  |
| No | 37778 (68.3) | 1171 (63.2) | 38949 (68.1) |  |
| **Endocrine treatment^+^** |  |  |  | 0.621 |
| Yes | 35586 (64.4) | 1204 (64.9) | 36790 (64.4) |  |
| No | 19712 (35.6) | 650 (35.1) | 20362 (35.6) |  |
| **Anti-HER2 therapy^+^** |  |  |  | 0.003 |
| Yes | 4918 (8.9) | 203 (10.9) | 5121 (9.0) |  |
| No | 50380 (91.1) | 1651 (89.1) | 52031 (91.0) |  |
| **Highest level of education** |  |  |  | 0.139 |
| ≤ 9 years | 12723 (23.0) | 461 (24.9) | 13184 (23.1) |  |
| 10-13 years | 23093 (41.8) | 762 (41.1) | 23855 (41.7) |  |
| > 13 years | 18894 (34.2) | 607 (32.7) | 19501 (34.1) |  |
| Missing | 588 (1.1) | 24 (1.3) | 612 (1.1) |  |
| **Family income** |  |  |  | 0.044 |
| Low | 13691 (24.8) | 506 (27.3) | 14197 (24.8) |  |
| Middle | 27661 (50.0) | 897 (48.4) | 28558 (50.0) |  |
| High | 13860 (25.1) | 447 (24.1) | 14307 (25.0) |  |
| Missing | 86 (0.2) | 4 (0.2) | 90 (0.2) |  |
| **Country of birth** |  |  |  | 0.256 |
| Sweden | 47509 (85.9) | 1568 (84.6) | 49077 (85.9) |  |
| Europe, not Sweden | 5297 (9.6) | 194 (10.5) | 5491 (9.6) |  |
| Outside of Europe | 2455 (4.4) | 91 (4.9) | 2546 (4.5) |  |
| Missing | 37 (0.1) | 1 (0.1) | 38 (0.1) |  |
| **CCI** |  |  |  | <0.001 |
| 0 | 47370 (85.7) | 1464 (79.0) | 48834 (85.4) |  |
| ≥1 | 7928 (14.3) | 390 (21.0) | 8318 (14.6) |  |

Figures in brackets are percentages if not marked by *. * implies that figures represent median value with (minimum, maximum).

^from preoperative core needle biopsy in case of neoadjuvant systemic treatment, from surgical specimen in case of primary surgery

**^+^**treatment received in the adjuvant and/or neoadjuvant setting

Abbreviations: BCS breast conserving surgery; IBR Immediate breast reconstruction; SLNB sentinel node biopsy; ALND axillary lymph node biopsy; NAT neoadjuvant therapy; CCI Charlson comorbidity index

**Table S4.** Odds ratios of least one major surgical postoperative complication within 30 days after first surgery as the binary outcome variable. Multivariable model is adjusted for all variables in the table.

|  |  | **Univariable** | | **Multivariable** | | |
| --- | --- | --- | --- | --- | --- | --- |
|  | **N (events)** | **OR (95% CI)** | ***P* value** | **OR (95% CI)** | ***P* value** |  |
| **Age (years)** |  |  |  |  |  |  |
| <40 | 2218 (85) | 1.29 (1.01-1.61) | 0.032 | 1.01 (0.79-1.28) | 0.940 |  |
| 40-49 | 8178 (301) | 1.23 (1.07-1.42) | 0.003 | 1.11 (0.96-1.29) | 0.148 |  |
| 50-64 | 20083 (603) | 1.00 (ref) |  | 1.00 (ref) |  |  |
| 65-74 | 17428 (494) | 0.94 (0.84-1.06) | 0.335 | 0.94 (0.82-1.06) | 0.307 |  |
| 75 and more | 9245 (371) | 1.35 (1.18-1.54) | <0.001 | 1.01 (0.86-1.18) | 0.931 |  |
| **Year of surgery** |  |  |  |  |  |  |
| 2008-2009 | 10778 (337) | 0.97 (0.82-1.15) | 0.737 | 0.87 (0.73-1.04) | 0.123 |  |
| 2010-2011 | 12627 (381) | 0.94 (0.79-1.11) | 0.436 | 0.89 (0.75-1.06) | 0.188 |  |
| 2012-2013 | 13103 (447) | 1.06 (0.91-1.25) | 0.460 | 1.03 (0.87-1.22) | 0.765 |  |
| 2014-2015 | 13462 (458) | 1.06 (0.90-1.25) | 0.479 | 1.04 (0.89-1.23) | 0.607 |  |
| 2016-2017 | 7182 (231) | 1.00 (ref) |  | 1.00 (ref) |  |  |
| **Region of residence** |  |  |  |  |  |  |
| Stockholm/  Gotland | 12642 (491) | 1.00 (ref) |  | 1.00 (ref) |  |  |
| Uppsala/Örebro | 12246 (339) | 0.70 (0.61-0.81) | <0.001 | 0.72 (0.62-0.84) | <0.001 |  |
| North | 4981 (203) | 1.05 (0.89-1.24) | 0.556 | 1.08 (0.90-1.29) | 0.395 |  |
| South | 10814 (296) | 0.70 (0.60-0.81) | <0.001 | 0.71 (0.60-0.82) | <0.001 |  |
| Southeast | 5859 (175) | 0.76 (0.64-0.91) | 0.009 | 0.73 (0.61-0.88) | 0.001 |  |
| West | 10610 (350) | 0.84 (0.73-0.97) | 0.017 | 0.83 (0.72-0.97) | 0.017 |  |
| **Breast surgery** |  |  |  |  |  |  |
| BCS | 33170 (767) | 1.00 (ref) |  | 1.00 (ref) |  |  |
| Mastectomy | 22388 (970) | 1.91 (1.74-2.11) | <0.001 | 1.68 (1.50-1.87) | <0.001 |  |
| Mastectomy + IBR | 1594 (117) | 3.35 (2.72-4.08) | <0.001 | 2.80 (2.24-3.47) | <0.001 |  |
| **Axillary surgery** |  |  |  |  |  |  |
| SNLB | 37191 (993) | 1.00 (ref) |  | 1.00 (ref) |  |  |
| ALND | 18556 (797) | 1.64 (1.49-1.80) | <0.001 | 1.38 (1.24-1.54) | <0.001 |  |
| **Highest level of education** |  |  |  |  |  |  |
| ≤ 9 years | 13184 (461) | 1.00 (ref) |  | 1.00 (ref) |  |  |
| 10-13 years | 23855 (762) | 0.91 (0.81-1.02) | 0.119 | 0.96 (0.85-1.09) | 0.545 |  |
| > 13 years | 19501 (607) | 0.89 (0.78-1.00) | 0.056 | 0.91 (0.79-1.05) | 0.185 |  |
| **Family income** |  |  |  |  |  |  |
| Low | 14197 (506) | 1.00 (ref) |  | 1.00 (ref) |  |  |
| Middle | 28558 (897) | 0.88 (0.79-0.98) | 0.021 | 0.96 (0.85-1.09) | 0.569 |  |
| High | 14307 (447) | 0.87 (0.77-0.99) | 0.039 | 0.93 (0.80-1.09) | 0.355 |  |
| **Country of birth** |  |  |  |  |  |  |
| Sweden | 49077 (1568) | 1.00 (ref) |  | 1.00 (ref) |  |  |
| Europe, not Sweden | 5491 (194) | 1.11 (0.95-1.29) | 0.179 | 1.06 (0.90-1.24) | 0.447 |  |
| Outside of Europe | 2546 (91) | 1.12 (0.90-1.38) | 0.290 | 0.97 (0.76-1.22) | 0.777 |  |
| **CCI** |  |  |  |  |  |  |
| 0 | 48834 (1464) | 1.00 (ref) |  | 1.00 (ref) |  |  |
| 1 | 2934 (126) | 1.45 (1.20-1.74) | <0.001 | 1.35 (1.10-1.64) | 0.003 |  |
| 2 | 1177 (54) | 1.56 (1.16-2.03) | 0.002 | 1.41 (1.03-1.88) | 0.025 |  |
| 3-5 | 725 (30) | 1.40 (0.95-1.98) | 0.076 | 1.37 (0.91-1.98) | 0.110 |  |
| 6-7 | 3125 (161) | 1.76 (1.48-2.07) | <0.001 | 1.20 (0.98-1.45) | 0.069 |  |
| 8 and more | 357 (19) | 1.82 (1.10-2.81) | 0.012 | 1.12 (0.63-1.83) | 0.675 |  |
| **Primary treatment** |  |  |  |  |  |  |
| Surgery | 54149 (1715) | 1.00 (ref) |  | 1.00 (ref) |  |  |
| NAT | 3003 (139) | 1.48 (1.24-1.76) | <0.001 | 0.85 (0.68-1.04) | 0.115 |  |

OR; odds ratio; CI; confidence interval; BCS breast conserving surgery; IBR Immediate breast reconstruction; SNLB sentinel node biopsy; ALND axillary lymph node biopsy; NAT neoadjuvant therapy
