# Supplementary figures and images for: Major surgical postoperative complications and survival in breast cancer: Swedish population-based register study in 57 152 women
Source: Br J Surg. 2022 Aug 5;109(10):977–83. doi: 10.1093/bjs/znac275 (PMC10364684; doi:10.1093/bjs/znac275)

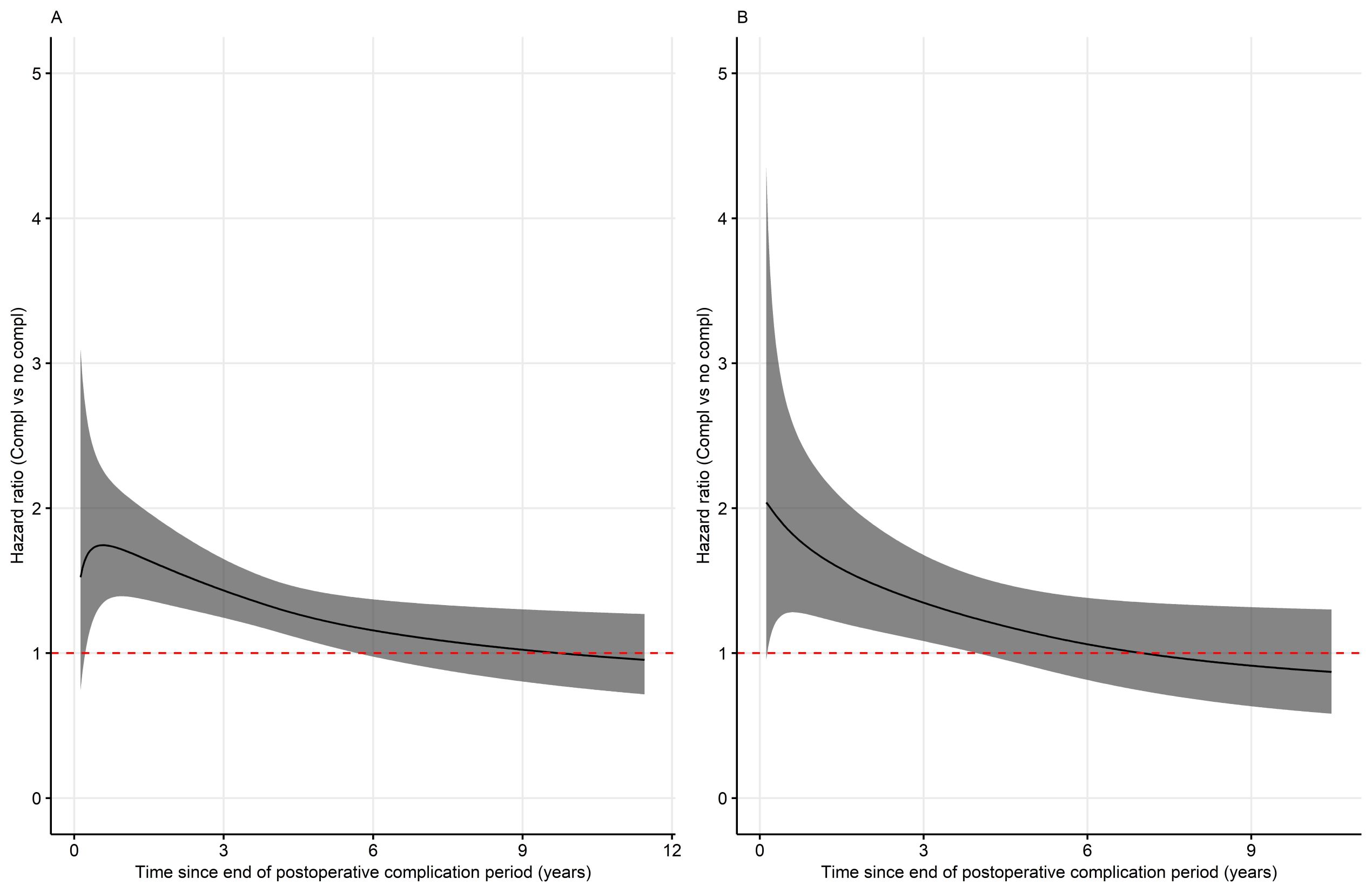

Supplement: znac275_Supplementary_Data [file znac275_supplementary_data.zip › Supplementary_Figure_2.jpg]
